# Resolution of unilateral sensorineural hearing loss in a pediatric patient with a severe phenotype of Muckle-Wells syndrome treated with Anakinra: a case report and review of the literature

**DOI:** 10.1186/s40463-018-0256-0

**Published:** 2018-01-30

**Authors:** Cinzia Marchica, Faisal Zawawi, Dania Basodan, Rosie Scuccimarri, Sam J. Daniel

**Affiliations:** 1 Department of Pediatric Otolaryngology Head and Neck Surgery, McGill University Health Center, Montreal Children’s Hospital, 1001, boul. Décarie - Local A02.3017, Montreal, QC H4A 3J1 Canada; 2 Division of Pediatric Rheumatology, McGill University Health Center, Montreal Children’s Hospital, 1001 Decarie, Montreal, Quebec Canada

**Keywords:** Muckle-wells syndrome, Hearing loss, Anakinra

## Abstract

**Background:**

Muckle-Wells syndrome (MWS) is a rare auto-inflammatory disease characterized by the presence of recurrent urticaria, deafness and amyloidosis. Progressive sensorineural hearing loss (SNHL) is reported to occur in up to 85% of patients occurring in the second and third decades and as early as the first decade in patients with a more severe phenotype, thus potentially having a significant impact on a child’s development. IL-1 inhibitors, such as Anakinra, have been described to improve systemic inflammation, and stabilize or improve hearing status as well. However, complete resolution of hearing loss has been rarely reported. The objective of this article is to highlight the clinical presentation of a pediatric patient with a severe form of MWS and report on the complete resolution of SNHL with the use of Anakinra.

**Case presentation:**

A 3-year-old boy was referred to our hospital to assess for the possibility of MWS given a history of hives and recurrent episodes of fever with a family history of MWS in his mother. Of note, the patient’s history was significant for conductive hearing loss, speech delay, as well as recurrent acute otitis media episodes. Genetic analysis was performed and diagnosis of MWS was confirmed due to the presence of a NLRP3 gene mutation. Further work-up demonstrated the presence of papilledema and elevation of systemic inflammatory markers for which Canakinumab was initiated. Despite initiation of this treatment, audiogram evaluation demonstrated a new right-sided SNHL. Lumbar puncture also revealed aseptic meningitis. Canakinumab was eventually discontinued and Anakinra initiated. Within 7 months of treatment with Anakinra at 5 mg/kg sc daily, resolution of the SNHL was observed. With further escalation of the Anakinra dose, there was also complete resolution of the aseptic meningitis.

**Conclusions:**

Progressive hearing loss is a significant finding in patients with MWS. Early screening as well as initiation of Anakinra can lead to complete resolution of SNHL even in a patient with a severe spectrum of MWS. However, as this case demonstrates, longer treatment duration and higher doses of Anakinra may be required to achieve this.

## Background

Muckle-Wells syndrome (MWS) is a rare auto-inflammatory disease following an autosomal dominant inheritance pattern [1]. It was first described in 1962 by Muckle and Wells and is characterized by the presence of recurrent urticaria, deafness and amyloidosis [2, 3]. In addition, patients can have fever, fatigue, conjunctivitis, headache, arthralgias/arthritis and raised inflammatory markers [1, 3]. MWS is part of a spectrum of cryopyrin-associated periodic syndromes (CAPS) which also includes two other clinical phenotypes, the less severe ‘familial cold auto-inflammatory syndrome’ (FCAS), and the more severe ‘neonatal-onset multisystem inflammatory disease’ (NOMID) or ‘chronic infantile neurological cutaneous articular syndrome’ (CINCA) [1, 3, 4]. NOMID/CINCA usually has more severe ocular, musculoskeletal and central nervous system (CNS) manifestations than MWS [4, 5]. The CNS manifestations of NOMID/CINCA include aseptic meningitis and increased intracranial pressure [5, 6]. However, the spectrum of CAPS is a clinical continuum with intermediate or overlapping forms [7, 8]. Progressive sensorineural hearing loss (SNHL) is reported to occur in up to 85% of patients with MWS and usually develops in the second or third decade of life [7] unlike NOMID/CINCA that usually occurs in the first decade [5].

CAPS has been associated with mutations in the NLRP3 gene encoding for the cryopyrin/NLRP3 protein, which has been shown to be fundamental in the activation of intracellular caspase 1 and the processing of interleukin-1ß (IL-1 ß) [4, 8, 9]. The management of CAPS patients has therefore been directed towards the use of IL-1 inhibitors. Canakinumab is a fully human, recombinant IgG1 anti–IL-1ß monoclonal antibody that selectively prevents IL-1ß from interacting with IL-1 receptors [8, 9]. Anakinra is a recombinant, non-glycosylated IL-1 receptor antagonist that has been demonstrated to inhibit the activity of IL-1 by binding competitively to the IL-1 type I receptor [1, 8, 9].

A number of studies in the literature have shown successful treatment of both the systemic symptoms and inflammation with either of the IL-1 inhibitors in CAPS [8]. In addition, hearing loss (HL) has improved or stabilized with anti-IL1 inhibitors in CAPS [3, 6, 9–20] however, complete resolution has been rarely reported [15, 16, 19, 20].

The objective of this article is to highlight the clinical presentation of a pediatric patient with MWS and to report on the complete resolution of HL in this child with the use of Anakinra.

This case report was conducted in the tertiary healthcare center of the Montreal Children’s Hospital of the McGill University Health Centre (MUHC). Ethical approval by the research ethics board (REB) of the MUHC was obtained. After appropriate consent of the patient as well as legal guardians (patients’ parents), a review of the patient’s medical records, radiological imaging, lab tests and operative reports was performed. A comprehensive literature review assessing the effect of Anakinra on hearing in CAPS was performed.

## Case presentation

A 3-year-old boy was referred to the Montreal Children’s Hospital to assess for the possibility of MWS given a history of hives and recurrent episodes of fever observed shortly after birth and the recent diagnosis of this syndrome in the child’s mother.

The patient had a past medical history of asthma. He had also received bilateral pressure equalizing tubes (PET) in the context of conductive HL, speech delay, presence of middle ear effusion and 4 episodes of acute otitis media in one year.

The patient’s mother was suspected of having MWS when amyloid was identified on small bowel biopsy as part of an evaluation for chronic diarrhea and weight loss. She also had nephrotic range proteinuria. Her history revealed that she had an urticarial rash since infancy and had later developed SNHL. Genetic analysis confirmed that she was heterozygous for D305N mutation in the NLRP3 gene. Given these results, her son was referred to the rheumatology as well as the medical genetics clinics of the Montreal Children’s Hospital. Genetic blood analysis confirmed the presence of the NLRP3 gene mutation and thus the diagnosis of MWS in this boy.

The child underwent a complete workup. His inflammatory markers were elevated: C-Reactive Protein (CRP) at 72 mg/L (normal 0–5) and Erythrocyte Sedimentation Rate (ESR) at 45 mm/h (normal 0–10). He had a normal complete cell count, normal creatinine and normal urinalysis with normal urine protein/creatinine ratio. An initial eye exam demonstrated the presence of papilledema which was thought to be most likely secondary to this syndrome. Magnetic resonance imaging (MRI) of the head was performed and it did not reveal any abnormalities or enhancing lesions.

The patient was started on Canakinumab in April 2012 at a dose of 50 mg (~ 2 mg/kg) subcutaneous (sc) every 8 weeks. The patient’s mother was also started on Canakinumab, however was quickly switched to Anakinra given persistent enhancement of the cochlea on MRI. A referral was made for evaluation for both child and mother at the National Institute of Health (NIH) and they were seen in October 2012. Following this, the patient was co-managed by both teams. In November 2012, the child’s dose of Canakinumab was changed to every 6 weeks because of persistent mildly elevated inflammatory markers (CRP 4.9–7.5; ESR 41). An effective response was observed with this change including normalization of the CRP and resolution of the papilledema.

In March 2013, the patient began having breakthrough symptoms of urticarial rash, as well as reappearance of an elevated CRP and ESR. The dose of Canakinumab was thus increased to 75 mg (5 mg/kg) every 6 weeks. Over the next few months, the patient improved with normalization of the inflammatory markers.

**Fig. 1:**
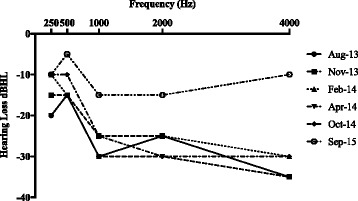
Audiogram results evaluating air conduction thresholds on the right ear for various assessments between August 2013 and September 2015. Prior to the initiation of Anakinra, SNHL was seen. A complete resolution of the hearing loss was seen 7 months following the initiation of Anakinra

In July 2013, one year after starting Canakinumab, audiogram evaluation demonstrated new right-sided mild SNHL with a possible mixed component (Fig. 1). Speech reception thresholds remained good at 15 decibels of HL (dBHL) in the left ear and 20 dBHL on the right ear. Tympanometry results displayed left sided large volumes compatible with a PET in place, however, negative pressure were observed on the right side.

At that time, MRI was repeated and continued to be normal with no enhancement of the cochlea or meninges. A lumbar puncture (LP) was done, which revealed the presence of aseptic meningitis with 19 white blood cells (WBCs) and an elevated protein count of 24 mg/dl in the cerebrospinal fluid (CSF). These occurred in the context of the child feeling well with no headaches, rash or fever and a normal CRP at 0.5 mg/L. The SNHL and abnormal LP results prompted an increase in the dose of Canakinumab to 150 mg every 6 weeks (8 mg/kg).

Follow up in otolaryngology and audiology continued to demonstrate mild SNHL on the right side. Approximately 7 months following the increase of Canakinumab to 8 mg/kg, the patient was still experiencing difficulties at school, perhaps secondary to hearing impairments. A repeat audiogram continued to demonstrate mild SNHL in the right ear from 750 Hz to 4000 Hz (Fig. 1). The left ear hearing remained within normal limits, except for a mild conductive HL at 2 kHz. Speech reception thresholds were mildly elevated in the right ear at 25 dBHL. Given this HL and aforementioned difficulty in school performance, the patient received a hearing aid for the right ear.

In May 2014, repeat LP showed persistent elevation in WBC at 13 (20% lymphocytes; 44% neutrophils) and protein at 17. The SNHL remained stable. At that time, the inflammatory markers remained normal (CRP 0.7; ESR 8). Due to the persistence of pleocytosis in the CSF and SNHL, in June 2014, Canakinumab was discontinued and the patient was started on Anakinra 100 mg sc daily (5 mg/kg).

**Fig. 2:**
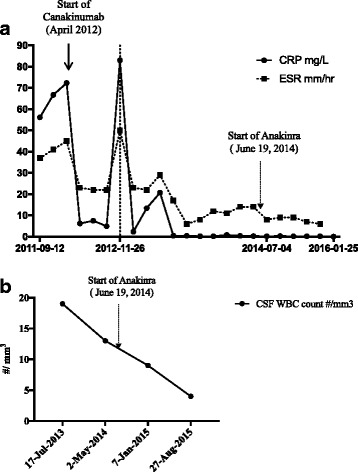
**a** Inflammatory Markers with time. C-Reactive Protein (CRP) and Erythrocyte Sedimentation Rate (ESR) were measured in time. This demonstrates the changes in the inflammatory markers throughout the disease course as well as the response to Canakinumab and Anakinra. **b** Cerebrospinal Fluid White blood cell counts. The White blood cell counts (WBC) in the Cerebrospinal fluid (CSF) of our patient. This demonstrates a significant reduction and normalization with Anakinra

By January 2015, within 7 months of initiating Anakinra, he had improving aseptic meningitis with CSF WBC’s decreasing to 9/mm^3^. The dose of Anakinra was increased further to 142 mg (6.5 mg/kg). Interestingly, at that time, from an otologic perspective, resolution of the HL was observed at the NIH and confirmed in follow-up at our center in September 2015. Audiograms demonstrated hearing that was within normal limits bilaterally, with normal otoacoustic emissions. When comparing to previous, a 20 dB improvement was seen spanning the 1000-4000 Hz frequency range (Fig. 1). Within another 7 months of the increase in dose of Anakinra, complete resolution of the aseptic meningitis was also observed with a CSF WBC count at 4/mm^3^ (Fig. 2a, b).

The patient continues to be treated with Anakinra at 6 mg/kg, and at the time of this publication, there has been no relapse of the HL or aseptic meningitis. He continues to be clinically well and persists with normal inflammatory markers and urinalysis.

## Discussion and conclusions

Knowledge on MWS has been continuously evolving since the identification of the linkage to the NLRP3 gene mutation. Amyloidosis and HL remain important complications of this disease [3]. SNHL in MWS is often rapidly progressive [3]. It affects primarily high frequencies of ≥ 6 kHz but can progress to complete deafness [3]. Earlier age at diagnosis was noted to be significantly associated with HL in one cohort study [3]. However, HL clearly increases with age [7, 12, 21]. Given its progressive nature, early diagnosis and treatment is imperative. Current therapeutic interventions include the use of anti-IL-1 therapy such as Anakinra or Canakinumab.

This case report describes a pediatric patient with a severe spectrum of MWS with likely an intermediate or overlapping phenotype with CINCA/NOMID given the papilledema and aseptic meningitis, who had complete resolution of HL with Anakinra. To our knowledge, this is the first pediatric case describing complete resolution of SNHL associated with a severe phenotype of MWS back to baseline normal hearing with Anakinra monotherapy.

Various studies have demonstrated Anakinra’s effectiveness in reducing the inflammatory manifestations seen in MWS [1, 9–11, 13, 14, 16, 19] including improvements in CRP, Amyloid A serum protein and systemic symptoms. In addition, Anakinra has also been shown to improve or stabilize HL in this syndrome [3, 10–14]. However, complete resolution of HL with Anakinra has only been described in 4 patients in the literature: two adults with MWS [16, 20], an 8 year old with MWS [19] and an adolescent with NOMID/CINCA [15].

Rynne and colleagues described a 15–30 dB improvement, confirmed from 250 Hz – 4000 Hz, in a 59-year-old woman treated with Anakinra for MWS [20]. This significant improvement allowed for the patient to no longer require hearing aids. However, audiograms still depicted a SNHL, which the authors believe to be secondary to presbyacusis [8]. Similarly, Mirault et al. described a 22-year-old female treated for MWS with bilateral SNHL since the age of 12 [16]. Three months following the initiation of Anakinra, repeat audiogram demonstrated a near-complete regression of the deafness, which remained stable after 18 months [16]. The difference with our case was that our patient had a more severe phenotype given the CNS symptoms. In addition, despite our patient having an earlier initiation of treatment, he required a longer treatment period before HL resolved. In an 8-year-old female, Anakinra was started shortly after detection of HL and resolution with treatment occurred within 3 months [19]. Unlike our case, this patient did not have CNS involvement and thus had a milder phenotype. The last case of complete HL resolution was that of a 14-year-old boy with NOMID/CINCA. This adolescent had moderate to severe HL requiring hearing aids and significant CNS symptoms refractory to multiple treatments [15]. Nine months after the introduction of Anakinra, in combination with other therapeutic agents, he had complete resolution of the HL [19]. However, in this case, the HL was described as conductive [19]. This case, similarly to our case, illustrates that in the more severe phenotypes, a longer treatment period may be required prior to HL improvement. Dalgic and colleagues evaluated the effect of Anakinra on a 14 year old with MWS [13]. Once again, a 10–20 dB improvement in HL was observed after 2 months of Anakinra treatment [13]. However, a mild-moderate HL persisted [13]. It is possible that complete reversal of HL would have occurred with a longer treatment period.

A number of cohort studies reveal varied results with treatment with Anakinra for MWS and CINCA/NOMID syndromes. Goldbach-Mansky et al. presented 18 pediatric patients with NOMID with varying degrees of HL [6]. Six of these patients (33%) showed hearing improvement by 6 months, and 9 patients (50%) had stable hearing relative to baseline with Anakinra [6]. In a cohort study of 13 patients with MWS, 92% presented with HL observed as young as 6 years old [11]. In addition, hearing improved in 3 of the 12 patients: one with Canakinumab and two with Anakinra [11]. In a cohort of 33 patients with MWS aged from 3 to 75 years, 67% had SNHL [12]. Hearing remained stable in a majority of patients with anti-IL-1 therapy, improved in 3 patients on Canakinumab and in 2 patients with Anakinra [12]. One patient’s HL worsened on Anakinra [12].

In a cohort study of 23 patients with MWS, SNHL was identified in 91% of patients [3]. In these, all had high frequency abnormalities including those with early HL and only 74% had abnormal standard assessments (0.5–4 kHz) [3]. A total of 44/46 (96%) of patient ears showed improvement or stable hearing with anti-IL-1 treatment [3]. Improvement was seen in 11 ears, 6 treated with Canakinumab and 5 with Anakinra [3]. Two ears worsened, both on Anakinra [3]. This group highlighted the importance of screening patients at high frequency hearing thresholds of 6 and 8 kHz, above the standard measurements of 4 kHz, given early high frequency HL in these patients [3].

In another study by Sibley et al., 26 patients with NOMID showed improvement in hearing in 30% of ears and stabilization of HL in the majority of patients [18]. Cochlear enhancement on MRI correlated with persistent HL [18]. In a cohort study of 12 patients with MWS, HL was present in 83% and improved in only 2 patients [14]. In one of these patients, a 15-year-old, had subjective resolution of the HL but the audiogram demonstrated persistent but improved mild impairment [14].

Finally, Neven et al. described 10 patients with NOMID/CINCA with HL reported in 7 of the 8 oldest patients [17]. With the initiation of Anakinra, only 2 patients (those with a previously documented moderate HL) demonstrated a 15 dB improvement following 6 months of treatment, with HL remaining stable in the others [17]. This paper suggested that residual CNS inflammation and deafness persisted in some patients, especially if there had been a delay in diagnosis and treatment [17].

In comparison to the above mentioned studies, our patient received an early diagnosis of MWS and subsequently commenced on treatment rapidly. Although initially started on Canakinumab, which demonstrated efficacy in reducing the inflammatory markers and the papilledema, the presence of aseptic meningitis as well as SNHL did not improve. Therefore, Canakinumab was stopped and Anakinra initiated. Following 7 months of treatment with Anakinra, resolution of the SNHL was observed; and with further escalation of the dose, the CSF finally normalized. Resolution of the HL may have occurred due to relatively early initiation of Anakinra. However, this case demonstrates that longer treatment duration and higher doses of Anakinra may be required to achieve this improvement.

In conclusion, early initiation of Anakinra can lead to complete resolution of SNHL even in a patient with a severe spectrum of MWS. Resolution of HL in this patient with Anakinra further supports the hypothesis that anti-IL1 therapy must be initiated promptly in order to limit the irreversible changes that may be brought on to the cochlea in this disorder.

## Abbreviations

CAPS: Cryopyrin-associated periodic syndromes
CINCA: Chronic infantile neurological cutaneous articular syndrome
CNS: Central nervous system
CRP: C-Reactive Protein
CSF: Cerebrospinal Fluid
dBHL: Decibels of HL
ESR: Erythrocyte Sedimentation Rate
FCAS: Familial cold auto-inflammatory syndrome
HL: Hearing loss
LP: Lumbar puncture
MRI: Magnetic resonance imaging
MWS: Muckle-Wells syndrome
NIH: National Institute of Health
NOMID: Neonatal-onset multisystem inflammatory disease
PET: Pressure equalizing tubes
sc: Subcutaneous
SNHL: Sensorineural Hearing Loss
WBC: White Blood Cells
